# Role of Myeloid-Epithelial-Reproductive Tyrosine Kinase and Macrophage Polarization in the Progression of Atherosclerotic Lesions Associated With Nonalcoholic Fatty Liver Disease

**DOI:** 10.3389/fphar.2019.00604

**Published:** 2019-05-29

**Authors:** Mirella Pastore, Stefania Grimaudo, Rosaria Maria Pipitone, Giulia Lori, Chiara Raggi, Salvatore Petta, Fabio Marra

**Affiliations:** ^1^Department of Experimental and Clinical Medicine, University of Florence, Florence, Italy; ^2^Section of Gastroenterology and Hepatology, PROMISE, University of Palermo, Palermo, Italy; ^3^Humanitas Clinical and Research Center, Rozzano, Italy

**Keywords:** monocytes, macrophages, nonalcoholic fatty liver disease, MerTK, inflammation, atherosclerosis, drug targeting

## Abstract

Recent lines of evidence highlight the involvement of myeloid-epithelial-reproductive tyrosine kinase (MerTK) in metabolic disease associated with liver damage. MerTK is mainly expressed in anti-inflammatory M2 macrophages where it mediates transcriptional changes including suppression of proinflammatory cytokines and enhancement of inflammatory repressors. MerTK is regulated by metabolic pathways through nuclear sensors including LXRs, PPARs, and RXRs, in response to apoptotic bodies or to other sources of cholesterol. Nonalcoholic fatty liver disease (NAFLD) is one of the most serious public health problems worldwide. It is a clinicopathological syndrome closely related to obesity, insulin resistance, and oxidative stress. It includes a spectrum of conditions ranging from simple steatosis, characterized by hepatic fat accumulation with or without inflammation, to nonalcoholic steatohepatitis (NASH), defined by hepatic fat deposition with hepatocellular damage, inflammation, and accumulating fibrosis. Several studies support an association between NAFLD and the incidence of cardiovascular diseases including atherosclerosis, a major cause of death worldwide. This pathological condition consists in a chronic and progressive inflammatory process in the intimal layer of large- and medium-sized arteries. The complications of advanced atherosclerosis include chronic or acute ischemic damage in the tissue perfused by the affected artery, leading to cellular death. By identifying specific targets influencing lipid metabolism and cardiovascular-related diseases, the present review highlights the role of MerTK in NAFLD-associated atherosclerotic lesions as a potential innovative therapeutic target. Therapeutic advantages might derive from the use of compounds selective for nuclear receptors targeting PPARs rather than LXRs regulating macrophage lipid metabolism and macrophage mediated inflammation, by favoring the expression of MerTK, which mediates an immunoregulatory action with a reduction in inflammation and in atherosclerosis.

## Metabolic Aspects of NAFLD: Insulin Resistance, Metabolic Syndrome, and Type 2 Diabetes

Nonalcoholic liver disease (NAFLD) was firstly described in 1980 (Ludwig et al., 1980) and is currently the most common cause of chronic liver disease worldwide (Li et al., 2018). The global prevalence of NAFLD is estimated to be approximately 25%, with the highest rates in South America (31%) and Middle East (32%), followed by Asia (27%), USA (24%), Europe (23%), and Africa (14%) (Younossi et al., 2016). NAFLD comprises a spectrum of conditions ranging from simple hepatic lipid accumulation without inflammation, defined nonalcoholic fatty liver or NAFL, to nonalcoholic steatohepatitis (NASH), characterized by hepatic fat deposition with hepatocellular damage, inflammation, and fibrosis. This latter form in a smaller proportion of patients may lead to a series of complications including cirrhosis, liver failure, and hepatocellular carcinoma (HCC) (Marra et al., 2008; Ofosu et al., 2018). Cirrhosis may develop after about 15–20 years of chronic hepatocellular damage, and it is mainly characterized by a modified deposition of extracellular matrix components that, in cirrhotic liver, can be up to six times higher than in normal liver (Parola and Pinzani, 2018). In addition, inflammatory response contributes to hepatic encephalopathy, portal hypertension, liver failure, and increased risk of HCC (Tacke and Trautwein, 2015).

The development and progression of NAFLD is a complex and multifactorial process. NAFLD pathogenesis was originally described by the “two-hits hypothesis” (Day and James, 1998). According to this assumption, the “first hit” is represented by an excess intrahepatic lipid accumulation due to high intake of saturated fats, obesity, IR, and excessive fatty acids in the circulation (Marra, 2004). This sensitizes the liver to further insults acting as a “second hit” (Del Campo et al., 2018) including oxidative stress, lipid peroxidation, and mitochondrial dysfunction. These events give rise to a lipotoxic microenvironment, which leads to further damage of the hepatic tissue, consequently promoting inflammation and fibrogenesis (Buzzetti et al., 2016). More recent investigation has hypothesized that appearance of NASH is the result of the effects of signals deriving from multiple sites, including the gut, the adipose tissue, the muscle, and the liver itself, as illustrated as the “multiple-hits” hypothesis (Tilg and Moschen, 2010). The mechanisms underlying liver fibrosis are intricate and involve the interplay of multiple factors. Among these, a key role is played by the cross-talk between various liver-resident and infiltrating cellular subsets, which produce and secrete different soluble mediators (cytokines and chemokines) (Weiskirchen et al., 2018). In most cases, tissue injury induces an inflammatory response involving the local vascular system, immune cells, and release of endocrine and neurological factors. In this context, non-parenchymal cells [endothelial and hepatic stellate cells (HSCs)] and resident or recruited immune cells [macrophages, dendritic cells (DCs), and mast cells] secrete a variety of pro-inflammatory molecules such as tumor necrosis factor-α (TNF-α) and interleukin-1β (IL-1β), pro-fibrotic factors including transforming growth factor-β (TGF-β) and pro-apoptotic mediators, as well as reactive oxygen species (Tilg and Diehl, 2000). All together, these signals lead to the activation of matrix-producing cells (including HSCs) and consequently to myofibroblast trans-differentiation (Weiskirchen et al., 2018).

NAFLD not only is related to obesity, hypertension, and inflammation but also is closely associated with insulin resistance (IR), metabolic syndrome (MetS), and type 2 diabetes (T2D) (Gentilini et al., 2016). A recent meta-analysis of published prospective studies has investigated for the first time the association between the presence of NAFLD and the risk of developing T2D and MetS. In particular, it has been observed that NAFLD (as diagnosed by either serum liver enzymes or ultrasonography) predicts T2D development over a median follow-up of 5 years in a pooled population of patients from 20 prospective studies. Moreover, NAFLD was also associated with an increase in MetS incidence over a median follow-up of 4.5 years in a pooled population of patients from eight prospective studies (Ballestri et al., 2016). Importantly, IR has been shown to be crucial for NAFLD progression. Indeed, approximately 80% of obese and diabetic patients are affected by NAFLD (Marchesini et al., 1999). IR is defined as the decreased ability of tissues to respond to insulin signals, and diverse types may be distinguished, a systemic and a hepatic insulin resistance. Systemic IR is characterized by the inability of insulin to diminish blood glucose levels in an appropriate manner due to the impairment of GLUT4 receptor translocation to the surface membrane of the muscle cell, leading to insulin-dependent lower glucose uptake (Petersen and Shulman, 2017). Hepatic IR is described by cessation of insulin-induced suppression of hepatic glucose production and increased stimulation of lipogenesis (Petersen and Shulman, 2017).

Interestingly, insulin also controls lipid metabolism, as it enhances fatty acid re-esterification into triglyceride in adipocytes and the liver. Metabolic actions of insulin are mediated by the PI3K–AKT/PKB pathway (Cohen, 2006), which is phosphorylated by the insulin receptor through two major substrates, insulin receptor substrate 1 and 2 (IRS-1 and IRS-2). Well-established AKT/PKB substrates include GSK-3, a glycogen synthesis regulator, FOXO transcription factors, which upon phosphorylation inhibit transcription of FOXO-dependent gluconeogenic genes (Carter and Brunet, 2007), and sterol regulatory element-binding protein 1c (SREBP-1c), thus enhancing expression of rate-limiting glycolytic and lipogenic enzymes (Foretz et al., 1999; Foufelle and Ferré, 2002).

Also, promoting *de novo* lipogenesis in the liver, mediated by SREBP-1c, IR inhibits lipid export in the form of triglyceride-rich very-low-density lipoprotein (VLDL), hepatic FFA oxidation, and triglyceride (TG) accumulation, the major form of lipids stored in NAFLD patients (Browning and Horton, 2004).

Liver is the principal site of lipid metabolism; hepatic necro-inflammation has a crucial atherogenic role because it exacerbates systemic IR and promotes atherogenic dyslipidemia, with increased triglycerides, decreased high-density lipoprotein (HDL)-cholesterol, and increased low-density lipoprotein (LDL)-cholesterol (Nobili et al., 2010). Moreover, increased levels of highly atherogenic small dense type A LDL-cholesterol and of oxidized LDL-cholesterol are frequently detected in NAFLD. The main alteration in atherogenesis is the TG hepatic overproduction of as well as cholesterol-enriched VLDL particles.

## NAFLD as a Risk Factor for Cardiovascular Diseases

NAFLD has been recognized as strong predictor of increased carotid intima-media thickness, independent of other known cardio-metabolic risk factors.

Hepatic fat accumulation may be an important determinant of the relationship between NAFLD and atherosclerosis. Recently, it has been proposed that fatty liver is not *per se* a risk factor for atherosclerosis, unless it is associated with metabolic derangements. It has been suggested that there might be two different forms of fatty liver disease: one mainly related to metabolic abnormalities and another due primarily to genetic factors, characterized by higher risk of progressive liver damage (Sookoian and Pirola, 2011; Hamaguchi et al., 2007).

NAFLD is associated with adverse metabolic and atherosclerosis risk profiles (Fox et al., 2007; Neeland et al., 2013). From the metabolic point of view, the biological mechanism responsible for NAFLD-associated atherogenesis could be due to the crosstalk between visceral adipose tissue (VAT), gut, muscle tissue, and liver (Tilg and Moschen, 2010). Indeed, expanded and inflamed VAT releases molecules, such as adipokines, IL-6, and TNF-α, potentially involved in IR and cardiovascular disease (CVD) development (Fargion et al., 2014). Moreover, dietary chylomicrons and *de novo* lipogenesis contribute to the increased hepatic FFA pool as well as the development of NAFLD (Kleiner and Brunt, 2012).

**Figure 1:**
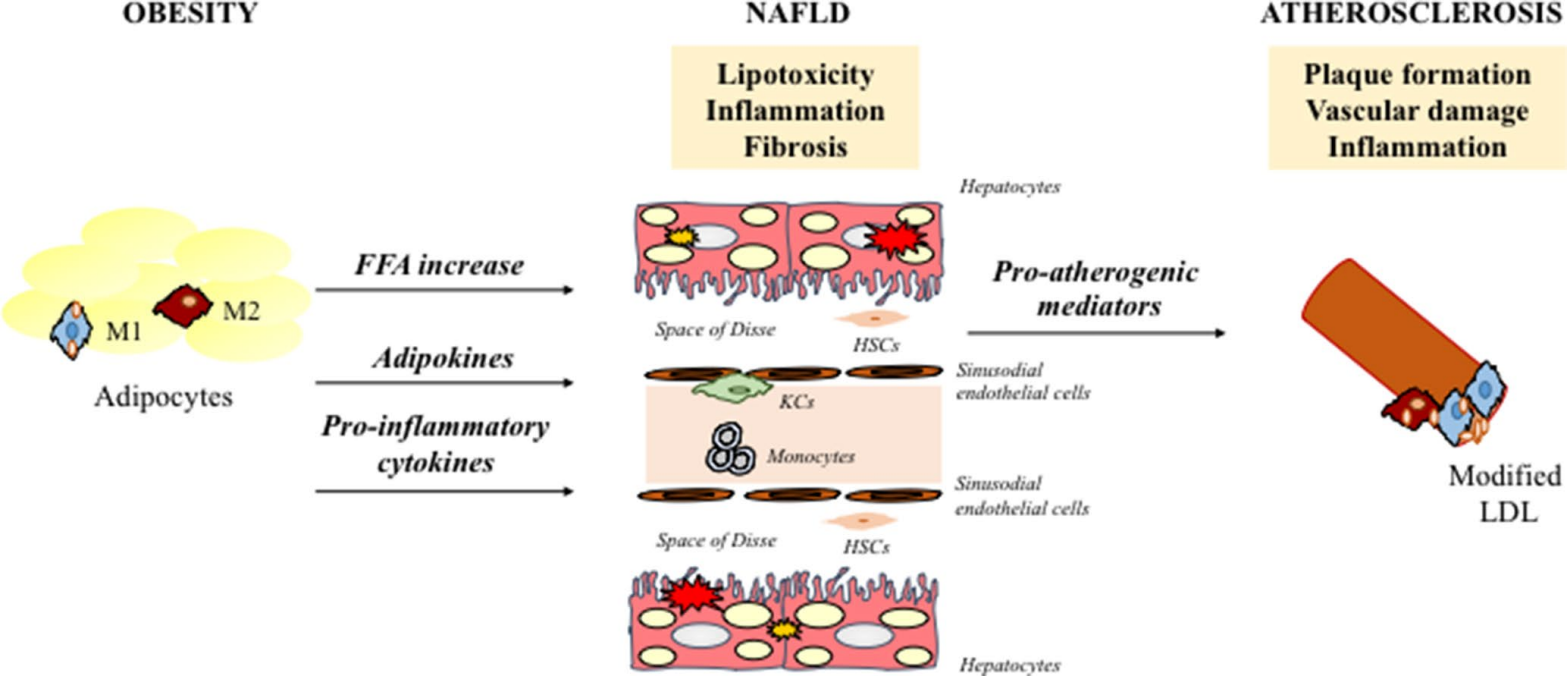
Schematic representation of key mechanisms responsible for NAFLD associated-atherosclerosis. NAFLD contributes to a more atherothrombotic risk profile via atherogenic dyslipidemia, hepatic/systematic insulin resistance and increased secretion of several proinflammatory and pro-coagulant mediators. NAFLD, nonalcoholic fatty liver disease; HSCs, hepatic stellate cells; FFA, free fatty acids; LDL, low-density lipoproteins; KCs, kupffer cells.

Lipid accumulation in the liver leads to sub-acute inflammation followed by cytokine production *via* the NF-kB pathway. In particular, the activation of NF-kB leads to increased transcription of several pro-inflammatory genes that mediate the progression of systemic and low-grade inflammation. The increase in adipose tissue and chronic inflammation also cause an imbalance in adipokine secretion, in particular a reduction of adiponectin. Adiponectin has been shown to have anti-inflammatory and anti-fibrotic capacity (Di Maira et al., 2018; Marra et al., 2008), and its low levels are associated with high fat content (Bugianesi et al., 2005) and the progression from steatosis and CVD to NASH and CV-atherosclerosis, respectively (Matsuzawa et al., 2004). NASH is involved in atherogenesis through the systemic release of pro-atherogenic mediators (C-reactive protein, IL-6, and TNF-α) and hypercoagulation and hypo-fibrinolysis induction mediated by fibrinogen, factor VII, and plasminogen-activator inhibitor-1 (Kotronen and Yki-Järvinen, 2008; Targher et al., 2008). In this way, the liver becomes a source of pro-atherogenic molecules that amplifies arterial injury. In line with these results, growing evidence indicates that atherosclerosis is proportional to the severity of liver damage (Alkhouri et al., 2010) (**Figure 1**).

The importance of NAFLD and its close association with CVD development has been highlighted by two meta-analyses. Notably, in a systematic meta-analysis of 34 cross-sectional and prospective cohort studies, an increase in coronary artery disease, hypertension, and atherosclerosis in NAFLD patients was observed, although no association between NAFLD/NASH and with overall or CVD-related mortality was shown (Wu et al., 2016). Additionally, (Targher et al., 2016) have described a strong correlation between NAFLD and increased risk of fatal and non-fatal CVD events, increased carotid intima-media thickness, increased coronary artery calcification, impaired flow-mediated vasodilation, and arterial stiffness. Indeed, several mechanisms correlated with NAFLD pathogenesis are involved in atherosclerosis and include genetic predisposition, reduced levels of adiponectin, IR, atherogenic dyslipidemia, oxidative stress, chronic inflammation, and altered production of pro- and anti-coagulant factors (Francque et al., 2016). Recently, a systematic review (Zhou et al., 2018) has described a two-fold increase in risk of CVD in diabetic NAFLD patients compared with non-NAFLD group, confirming other previous findings (Targher et al., 2007; Hamaguchi et al., 2007). Likewise, other two independent studies found that TG to high-density lipoprotein cholesterol ratio (TG/HDL-C) could be considered a better NAFLD predictor compared to other several lipid parameters and markers of liver injury (Ren et al., 2019; Fan et al., 2019).

Remarkably, also several polymorphisms associated with predisposition of NAFLD progression have been identified. Among the most validated factors, the Patatin-like phospholipase-3 (PNPLA3)/adiponutrin, rs738409 C > G SNP, I148M (Valenti et al., 2010) variant is involved both in hepatic lipid remodeling and in lipoprotein secretion, determining a greater predisposition to NASH (He et al., 2010; Ruhanen et al., 2014). Indeed, there is dissociation between *de novo* lipogenesis and the severity of hepatic steatosis in carriers of the I148M variant (Mancina et al., 2015). The involvement of the PNPLA3 variant has been observed also in lean subjects, where the presence of PNPLA3 GG genotype is correlated with a more severe liver and cardiovascular damage (Fracanzani et al., 2017). Among NAFLD patients, with minor metabolic alterations, the presence of GG PNPLA3 makes the subjects more susceptible to liver and CVDs, amplifying the effects of environmental factors (Fracanzani et al., 2017). In addition, carotid plaques have been independently associated not only with well-known risk factors for atherosclerosis but also with the PNPLA3 GG genotype (Petta et al., 2013).

Moreover, the transmembrane 6 superfamily member 2 (TM6SF2) rs58542926 C > T SNP, which encodes the loss of function E167K variant, has been associated with higher risk of NAFLD progression but with lower risk of cardiovascular events (Pirazzi et al., 2012). This protective effect of the E167K variant reflects the reduced circulating levels of atherogenic lipoproteins, because of higher intracellular lipid retention, mainly TGs and cholesterol, in hepatocytes. The mechanism seems related to the reduction of VLDL secretion, thus resulting in TG accumulation and consequent steatosis (Dongiovanni et al., 2015).

## Activation of Macrophages Depends on Their Metabolic State

In the liver, resident macrophages, the Kupffer cells are central players in the development of NASH by recruiting inflammatory immune cells and secreting pro-inflammatory cytokines (Sica et al., 2014; Raggi et al., 2015; Raggi et al., 2017). Importantly, the balance between M1 and M2 macrophages (**Box 1**) mediates the progression or resolution of liver fibrosis. Intriguingly, M1-M2 functional changes have been shown to be dependent on underlying metabolic changes (O’Neill and Pearce, 2016).

Box 1 **Macrophage polarization.**

In response to various signals, activated macrophages differentiate into two main subsets: M1 (classically activated) and M2 (alternatively activated). M1 macrophages are stimulated by LPS, INF-γ, TNF-α, and/or granulocyte macrophage colony-stimulating factor (GM-CSF) to produce inflammatory mediators, including TNF-α, IL-1β, IL-6, IL-8, IL-12, chemokine (C-C motif) ligand 2 (CCL2/MCP-1), reactive oxygen species (ROS), and reactive nitrogen intermediates (RNI), promoting inflammatory responses and HSC activation (Nathan, 2002). M2 macrophages regulate inflammatory reactions and tissue repair and can be distinguished in diverse subtypes, each one induced by different cytokines and eliciting different signals. In particular, M2a macrophages (CD206/mannose receptor^+^ CD209/DC-SIGN^+^ CD163^−^ CD16^−^ MerTK^−^) are stimulated by IL-4 and/or IL-13 and induce mainly a Th2 response. M2b macrophages are stimulated by immune complexes or LTR ligands and are involved in Th2 activation and immune regulation, producing IL-10 and inflammatory cytokines. Finally, M2c macrophages (CD206^high^ CD209^−^ CD163^+^ CD16^+^ MerTK^+^) are stimulated by macrophage colony-stimulating factor (M-CSF) plus IL-10 and TGF-β or by glucocorticoids, are characterized by their ability to secrete IL-10, which, in turn, is amplified by Gas-6 secretion in an autocrine manner, *via* MerTK signaling, and are involved in immune suppression, tissue repair, matrix remodeling, and clearance of apoptotic cells (Zizzo et al., 2012; Martinez et al., 2006).

**Figure:**
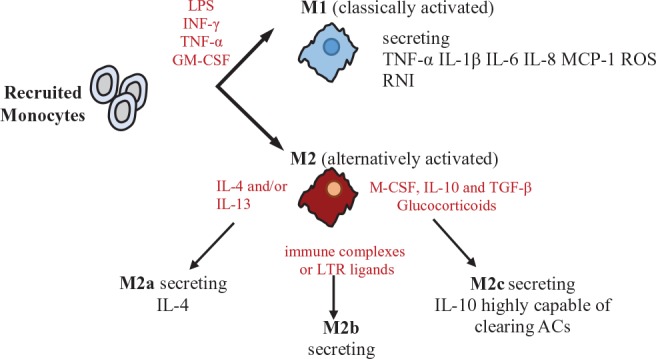

However, this concept is a little too simplistic to describe the polarization of liver macrophages, especially in pathological conditions. In the injured liver, macrophages often express markers of inflammation or resolution simultaneously, and can rapidly change their phenotype depending on the hepatic microenvironment (Tacke, 2017). During the early stages of liver injury, bone marrow-derived monocytes are intensively recruited to the liver and differentiate into inflammatory macrophages (mostly M1) to produce pro-inflammatory and profibrotic cytokines. Subsequently, recruited macrophages switch to an M2 phenotype, which secretes a wide variety of matrix metalloproteinases (MMPs), such as MMP-2, MMP-9, MMP-12, MMP-13, and MMP-14, and anti-inflammatory cytokines such as IL-4, IL-13, and IL-10, aimed to facilitate fibrosis resolution (Pradere et al., 2013).

In the lean adipose tissue, M2 macrophages produce high amounts of ATP (∼30 per glucose) through oxidative phosphorylation, a biochemical process slower than glycolysis (O’Neill et al., 2016). In contrast, increased lipid storage in obesity is associated with adipocyte dysfunction and a pro-inflammatory response, with an increase in M1-polarized pro-inflammatory macrophages (Norata et al., 2015). In particular, activation of immune receptors, such as TLRs, IL-1 receptor type I, and TNF-R, results in activation of NF-kB and JNK signaling, which can induce serine phosphorylation of IRS-1 and IRS-2 and thereby inhibition of downstream insulin signaling (McNelis and Olefsky, 2014; Marra, 2008).

**Figure 2:**
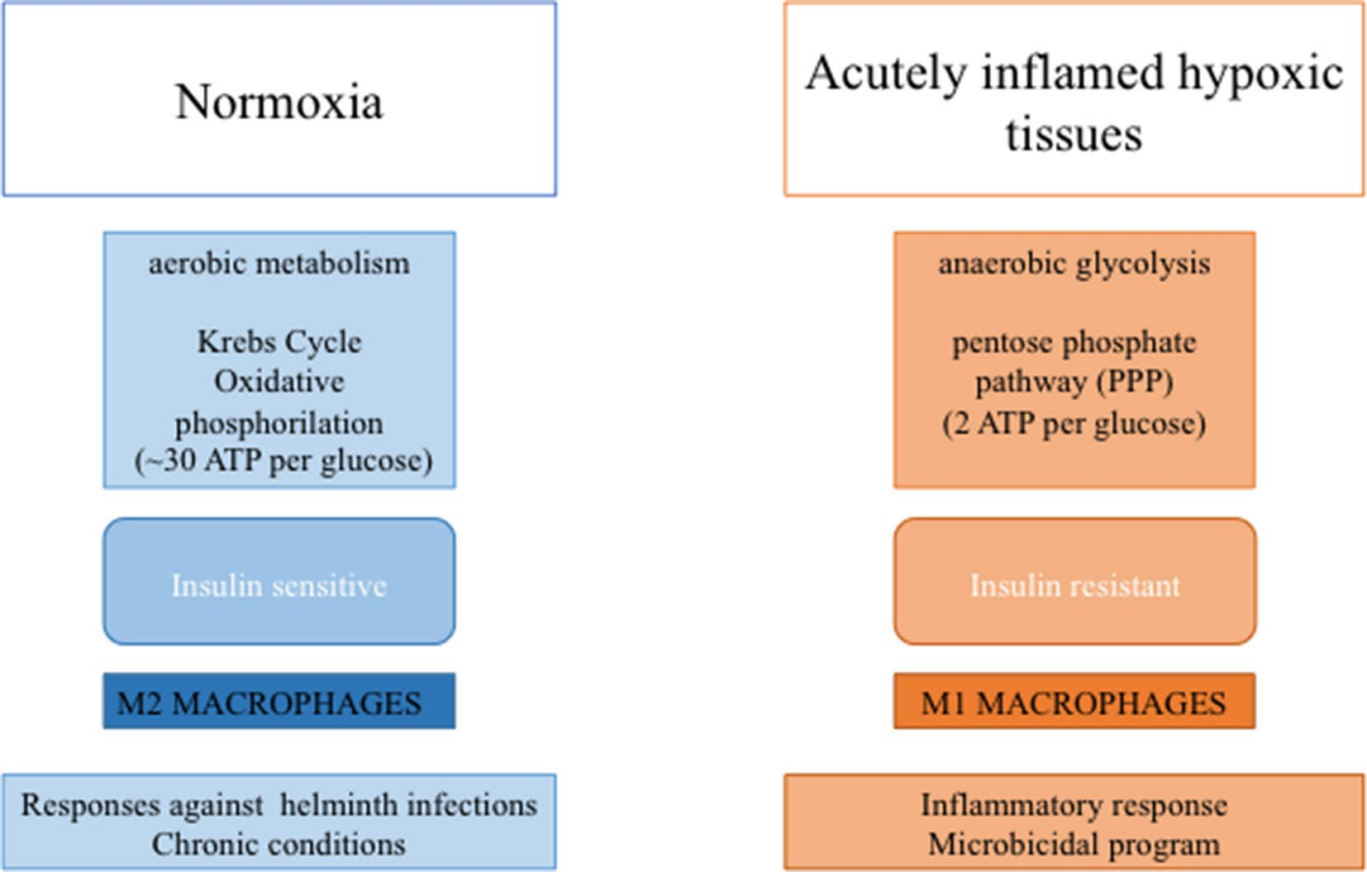
Macrophage-mediated functions to metabolic activities. In diagram on the left are depicted tissue features in the lean state associate with M2- macrophage polarization, while in diagram on the right are depicted tissue features during inflammation and insulin resistance associate with M1-macrophage polarization.

In M1 macrophages, upregulation of glycolytic metabolism feeds the pentose phosphate pathway (Van den Bossche et al., 2017). Although glycolysis produces only a small amount of energy (two molecules of ATP per glucose) (Nagy and Haschemi, 2015), this pathway supports inflammatory macrophage responses by generating NADPH, utilized by inducible NO synthase (iNOS) to produce NO or by NADPH oxidase to produce ROS, both necessary to sustain the antimicrobial activity of pro-inflammatory macrophages (Modolell et al., 1995). Moreover, glycolysis generates pyruvate to fuel the tricarboxylic acid cycle. In M1 macrophages, this cycle is interrupted after citrate and succinate (Jha et al., 2015; O’Neill, 2015). Increased synthesis of acetyl coenzyme A from citrate determines the synthesis of free fatty acids (FFAs), lipids, and prostaglandins (Infantino et al., 2011; Infantino et al., 2013). Up-regulation of proteins involved in the uptake [e.g., CD36 (Bassaganya-Riera et al., 2009)], esterification [e.g., diacylglycerol O-acyltransferase (Koliwad et al., 2010)], and oxidation [e.g., long-chain 3-hydroxyacyl-CoA dehydrogenase (Vats et al., 2006)] of FFAs could provide energy for M2 cells to restore tissue homeostasis (Shapiro et al., 2011). These processes would allow a reduction of FFA concentration by reducing IR and inflammation (Vats et al., 2006) (**Figure 2**).

## MerTK in the Activation of Anti-Inflammatory M2c Macrophages

MerTK represents the second member of Tyro-3, Axl, and Mer (TAM) receptor tyrosine kinase (RTK) family to be described (Linger et al., 2008).These receptors are characterized by adhesion molecule-like domains in the extracellular region, mimicking the structure of neural cell adhesion molecule important in cell–cell contacts, which contains five Ig domains and two fibronectin type III domains (Yamagata et al., 2003). The best-studied ligands for MerTK are the Vit-K modified-carboxylated proteins growth arrest-specific 6 (Gas-6) and Protein-S (Mark et al., 1996; Anderson et al., 2003). These glycoproteins share ∼44% of homology and have analogous domain structure, consisting of an N-terminal-carboxyl-glutamic acid (Gla) domain, four tandem epidermal growth factor (EGF)-like repeats, and a C-terminal sex hormone-binding globulin-like region (SHBG) containing 2 laminin G (LG) repeats (Mark et al., 1996). Gas-6 and Protein-S are biologically active following the carboxylation of the Gla-domain through a vitamin K-dependent reaction (Stenhoff et al., 2004). This domain mediates the Ca^2+^-dependent binding to negatively charged membrane phospholipids, such as phosphatidylserine exposed on the surface of apoptotic cells (Huang et al., 2003). LG domains are involved in the ligand–receptor interaction by forming a V-shaped structure, stabilized by a calcium-binding site (Sasaki et al., 2002). In human plasma, protein-S is highly concentrated (0.30 µM/L) (Rezende et al., 2004) (approximately 1,000 times higher), compared to Gas-6 (0.16–0.28 nM/L) (Balogh et al., 2005), conceivably due to the involvement of protein-S in the coagulation pathways, where it functions as a co-factor for protein C during factor Va and VIIIa inactivation (Heeb, 2008). Gas-6 is expressed mainly in vascular smooth muscle and endothelial cells, and it is frequently upregulated after tissue damage (Ekman et al., 2010).

MerTK is normally expressed in monocytes/macrophages, DCs, NK cells, NKT cells, HSCs, megakaryocytes, platelets, epithelial tissue, and reproductive tissue (Behrens et al., 2003; Petta et al., 2016). M2c macrophages express MerTK at high levels and display a marked capability to clear apoptotic bodies, a physiological process defined as efferocytosis (Zizzo et al., 2012). Interestingly, it has been demonstrated that M2c polarization is closely associated with MerTK upregulation, and detection of M2c receptors predicts MerTK expression (Zizzo et al., 2012). Indeed, MerTK expression and Gas-6 secretion follow the expression of specific M2c macrophages CD163 and CD16. This specific macrophage phenotype can be induced by M-CSF or dexamethasone, and IL-10 could enhance M-CSF effects. In addition, M2c macrophages are able to release Gas-6, which can, in turn, amplify IL-10 secretion in an autocrine manner, *via* MerTK signaling (Zizzo et al., 2012). Gas-6, linked to the externalized phosphatidylserine, activates MerTK, initiating the phagocytic process and inducing the activation of downstream pathways, such as ERK, P38, MAPK, FAK, AKT, and STAT-6, that mediate transcriptional events, leading to a decrease of pro-inflammatory cytokines, such as IL-12, and increase in inflammatory repressors, including IL-10 and TGF-β, thus generating an anti-inflammatory milieu (Tibrewal et al., 2008).

MerTK signaling plays a central role in the suppression of the innate immune response, as demonstrated in experimental models of endotoxemia, in which MerTK knockout mice exhibit an extreme activation of inflammatory responses and ineffective resolution of inflammation, mediated by elevated levels of TNF-α and IL-1 (Lee et al., 2012). MerTK acts by maintaining both central and peripheral tolerance, through different mechanisms, including efferocytosis. It is already well-described MerTK overexpression in murine models of fibrogenesis and in patients with NASH and severe fibrosis (Petta et al., 2016). Indeed, in genome-wide association studies, it has been reported that the MERTK locus rs4374383 G > A correlates with decreased hepatic MerTK expression, thus protecting against liver fibrosis in chronic hepatitis C and NAFLD (Patin et al., 2012; Petta et al., 2016). The same G > A variant has been found to be associated with cardio-metabolic derangement and nutritionally induced inflammation and could contribute in this way to liver and cardio-metabolic disease (Musso et al., 2017). Moreover, it has been shown that in human NAFLD specimens, MerTK is mainly expressed in macrophages and HSCs loosely aggregated within inflammatory foci (Petta et al., 2016). MerTK signaling has been recently studied also in humans with both acute liver failure syndromes and acute-on-chronic liver failure, where a significant cause of morbidity is sepsis. Triantafyllou et al. (Triantafyllou et al., 2018) have shown an expansion of MerTK-positive cells in circulatory and tissue compartments of patients with acute liver failure compared with healthy and cirrhotic controls, together with a concomitant increase in Gas-6 and in MerTK phosphorylation.

Notably, in response to acute liver injury, MerTK mediates downregulation of inflammatory cascades contributing to hepatic immune regulation by preventing autoreactive T cell development. However, in the context of chronic inflammation, MerTK promotes HSC activation, thus resulting in excessive fibrogenesis by abundant collagen and extracellular matrix proteins secretion (Petta et al., 2016).

Various therapies targeting MerTK are currently under development. Small-molecule tyrosine kinase inhibitors such as UNC569 (Christoph et al., 2013), UNC1062 (Schlegel et al., 2013), and UNC1666 (Lee-Sherick et al., 2015) have been recently described. These compounds competitively bind MerTK in its catalytic site, impeding phosphorylation and activation of the kinase domain. Treatment with these inhibitors causes a decrease in MerTK downstream signaling. Next-generation inhibitors have also been reported, including UNC2025, a potent, orally bioavailable inhibitor (Zhang et al., 2014). Other agents include Mer590, a monoclonal antibody that directly binds to the extracellular domain and induces internalization and degradation of MerTK (Cummings et al., 2014).

Small-molecule inhibitors and monoclonal antibodies are the main drugs that are currently used to inhibit signaling pathways by interfacing with specific molecules. Nevertheless, any targeted therapy has its own limitations. Although identifying a specific molecular target is crucial for NAFLD-associated cardiovascular treatment, targeting only a single molecule may not be completely determinant of these complex diseases. Other limitations include toxicity during the treatment, as well as mechanisms of resistance to molecular-targeted drugs.

## Macrophage Nuclear Receptors Control MerTK Expression in Lipid Metabolism NAFLD Associated

Macrophage polarization is an important mechanism for the regulation of inflammatory response, and it is finely controlled by the nuclear receptor superfamily members peroxisome proliferator activated receptors (PPARs) (α, β/δ, and γ isotypes) and liver X receptors (LXRs) (LXR-α and LXR-β) (Rigamonti et al., 2008). These transcription factors form heterodimers with retinoid X receptors (RXR) (α and β isotypes) and, upon binding a lipid or synthetic ligand, mediate gene expression through trans-activation (Szanto and Roszer 2008). Nuclear receptors have considerable roles in the modulation of macrophage functions. Their ligands influence the transcription of genes regulating lipid homeostasis, pro-inflammatory cytokine production, resolution of inflammation, and synthesis of mediators that promote tissue healing (Rőszer et al., 2013; Menendez-Gutierrez et al., 2012). PPAR-γ can be activated by metabolic signals (i.e., polyunsaturated fatty acids and lipoproteins) (Nagy et al., 1998), by inflammatory mediators (i.e., eicosanoids) (Kliewer et al., 1997), or by immunologic signals (i.e., cytokines) (Huang et al., 1999). PPAR-γ activation results in lipid uptake through the scavenger receptor CD36, and β-oxidation of fatty acids (Szanto and Roszer, 2008) is associated to macrophage polarization into M2a cells (Bouhlel et al., 2007). LXRs are oxysterol-activated transcription factors that sense elevated cellular cholesterol (Repa and Mangelsdorf, 2002). PPAR-γ and LXR activities are coordinated, PPAR-γ is in fact able to activate LXRs, but in certain conditions, PPAR-γ and LXRs exert opposing roles (Szanto and Roszer, 2008). In M2a macrophages, IL-4 stimulates the increase of PPAR-γ expression and LXR-α downregulation (Chinetti-Gbaguidi et al., 2011). LXRs are important for both apoptotic cell clearance and suppression of the inflammatory response during their phagocytosis. PPARs and LXRs control the transcription of many receptors, including MerTK.

Accumulation of excess lipoprotein-derived cholesterol in macrophages activates LXRs that, in turn, trigger the induction of ABC transporter, mediating cholesterol efflux (Castrillo and Tontonoz, 2004) and the upregulation of MerTK in mice (A-Gonzalez et al., 2009) and in humans (Zizzo and Cohen, 2015). Gonzalez et al. have demonstrated that phagocytosis of apoptotic cells activates LXRs, probably through the accumulation of membrane-derived cholesterol. LXRs, in turn, activate transcription of MerTK, generating a positive feedback to promote further efferocytosis, a process that mediates the increased expression of ABC transporter genes such as ABCA-1 and ABCG-1, involved in the efflux of the excess cholesterol and immunosuppression (A-Gonzalez et al., 2009). These results indicate that the LXR-dependent regulation of MerTK is important for normal immune homeostasis. MERTK^-/-^ and LXRs DKO mice share a series of features, including amplified pro-inflammatory responses and increased susceptibility to both autoimmunity and atherosclerosis (Ait-Oufella et al., 2008; Cohen et al., 2002).

## Role of MerTK in Atherosclerosis Process

Atherosclerotic lesions are clinically silent, and the acute cardiovascular events can be consequent to evolution to necrotic plaques (Virmani et al., 2000). At first, apoptotic cells are efficiently cleared by neighboring macrophages to limit overall lesion cellularity (Tabas, 2005). Here, efferocytosis is rapid and without inflammation. In physiological conditions, apoptotic cells are engulfed and degraded in phagolysosomes, and macrophages become overloaded with macromolecular constituents and cholesterol. In advanced atherosclerosis, the persistence of chronic inflammatory stimuli promotes lesion destabilization and susceptibility to heart attack and stroke. The role of inflammation in promoting atherosclerosis is well documented. Indeed, in advanced plaques, apoptotic foam cells, induced by chronic endoplasmic reticulum stress, elicit inflammatory responses (Li et al., 2013). In addition, endoplasmic reticulum stress is strongly correlated with plaque rupture (Li et al., 2006). Two processes contribute to post-apoptotic necrosis and defective efferocytosis and are impaired to resolve the inflammation response (Schrijvers et al., 2005; Libby, 2002; Tabas, 2010). Defective efferocytosis may be manifest at multiple levels, including improper presentation of apoptotic bodies ligands, failure to secrete *come find me* recruitment signals, or defects at the level of phagocytes (Vandivier et al., 2006). Efferocytosis is impaired in this last stage, and defective MerTK contributes, at least in part, to expansion of necrotic plaque (Tabas, 2005). In this regard, evidence demonstrates that mice lacking MerTK have shown a defect in efferocytosis, and this correlated with increased inflammation and necrosis within the plaque (Ait-Oufella et al., 2008; Thorp et al., 2008). Moreover, macrophages near the necrotic core of human atheroma showed lower MerTK expression than those in the peripheral lesions (Garbin et al., 2013). Finally, in the advanced atherosclerosis, accumulation of lipids and ROS increases levels of oxidized phospholipids. These lipids can bind to scavenger receptors and may compete for apoptotic cell recognition, compromising efferocytosis mechanisms (Gillotte-Taylor et al., 2001). A recent study shows that in the lesions, prevention of dead cells’ uptake is mediated by some apoptotic cells displaying a *don’t-eat-me* molecule called CD47, which is usually lost during apoptosis (Kojima et al., 2016).

Efferocytosis can be impaired by inactivation of MerTK under some inflammatory conditions (Sather et al., 2007). In particular, oxidized LDLs induce the expression of toll-like receptor 4 (TLR4), increase secretion of pro-atherogenic cytokines, such as TNF-α and IL-1β, and reduce secretion of anti-inflammatory cytokines, such as TGF-β and IL-10 (Bae et al., 2009). This pro-inflammatory environment impairs efferocytosis, promoting increased lipid uptake, which amplified phagocytosis, and reducing MerTK expression levels on the macrophage surface (Miller et al., 2003). The decrease of MerTK expression is associated with its cleavage by the metalloproteinase ADAM17. In human atheromas, macrophages adjacent to the necrotic core have higher ADAM17 than those in peripheral lesion (Garbin et al., 2013). Multiple athero-inflammatory stimuli, such as oxidative stress, hypoxia, and oxidized ligands, are able to promote ADAM17 activity (Sather et al., 2007; Garbin et al., 2013). Efferocytosis is suppressed by destroying the receptor and creating soluble Mer (sol-Mer), which competes for the binding molecules Gas-6 and Protein-S. Interestingly, oxidized LDLs, promoting MerTK cleavage and defective efferocytosis, can activate necroptotic pathways within advanced plaques, favoring the development of necrotic core (Karunakaran et al., 2016). In a recent study, it has been demonstrated that oxidized LDLs are able to increase sol-Mer levels and decrease MerTK expression in the surface of wild-type macrophages but not in macrophages pre-treated with ADAM17 inhibitor or in macrophages that express cleavage-resistant MerTK (Cai et al., 2016). Of note, MerTK-mediated efferocytosis might be limited by availability of Gas-6. In this regard, vascular smooth muscle cells appear to be a major source of Gas-6 within the lesions (Melaragno et al., 1999; Yin et al., 2000). It has been reported that vulnerable plaques have a paucity of smooth muscle cells in areas next to rupture (Clarke et al., 2006).

## Nuclear Receptors as Macrophage Therapeutic Target

Nuclear receptors such as PPARs and LXRs are important transcription factors associated with the specific accessory functions of macrophages. PPAR-γ exhibits great potentiality as a drug target in the therapy of inflammation-related diseases. Thiazolidinediones are insulin sensitizers used to improve glycemic control in T2DM patients. However, they may cause weight gain, fluid retention that can precipitate cardiac failure and bone fractures, and risk of bladder cancer (Cariou et al., 2012). In order to eliminate the onset of these effects, further research into new PPAR-γ modulators is required.

GW9662 is a potent, irreversible, and selective PPAR-γ antagonist. Zizzo et al. have shown that this PPAR-γ antagonist induces macrophage differentiation towards M2c-like (CD206^+^ CD163^+^ CD16^+^) cells and upregulation of the MerTK/Gas-6 axis. It has shown that among the novel small molecules derived from GW9662, BZ-26 has a stronger interaction with PPAR-γ and higher transcriptional inhibitory activity of PPAR-γ compared with GW9662. BZ-26 inhibits inflammatory macrophage differentiation of THP1 human monocytic cell line (Bei et al., 2016). Moreover, BZ-26 attenuates the inflammatory responses in LPS-triggered acute inflammation mouse model down-regulating peripheral TNF-α and IL-6 level. BZ-26 inhibits NF-kB transcriptional activity and abolishes LPS-induced nuclear translocation of P65. These data demonstrate that PPAR-γ, besides being a ligand-activated nuclear receptor implicated in regulation of lipid and glucose metabolism, is a fundamental transcription factor for differentiation and activation of macrophages. PPAR-γ could represent an important therapeutic target to modulate inflammation *via* inhibiting inflammatory macrophages.

LXRs are important regulators of cholesterol, free fatty acids, and glucose metabolism. LXRs drive cholesterol efflux in macrophages (through ABCA-1 and ABCG-1) and support reverse cholesterol transport by cholesterol conversion to bile acids and excretion in the liver. Moreover, their activation is important in regulating immune processes and in inhibiting inflammatory gene expression (Joseph et al., 2003). It has been shown that T0901317, a synthetic LXR agonist, upregulates MerTK expression during the polarization of monocytes to macrophages independently of M2c polarization, with significant effects already occurring at low doses (Zizzo and Cohen, 2015), confirming previously obtained data in mice (A-Gonzalez et al., 2009). Unfortunately, synthetic LXR agonists, such as T0901317, mediate reverse cholesterol transport not only in the macrophage but also in other cell types, including hepatocytes (Grefhorst et al., 2002). Activation of cholesterol efflux from both sources induces the activation of a lipogenic program, mediated by SREBP (Grefhorst et al., 2002), which induces remarkable steatosis and dyslipidemia in the liver in mouse models and human patients (Kirchgessner et al., 2016). These conditions make LXR’s therapeutic targeting unsustainable. Recently, Muse et al. have identified two synthetic compounds: N,N-dimethyl-3β-hydroxycholenamide (DMHCA) and methylpiperidinyl-3β-hydroxycholenamide (MePipHCA), which act as potent activators of LXR target genes involved in cholesterol efflux (e.g., ABCA-1 and ABCG-1) in human and murine macrophages, while having no effect on the expression of lipogenic SREBP targets (e.g., Fasn) in the liver (Magida and Evans, 2018). Interestingly, DMHCA and MePipHCA activity on Kupffer cells does not induce target gene activation in the liver (Muse et al., 2018). These two LXR agonists exhibit anti-atherosclerotic activity without causing substantial hypertriglyceridemia in mice; therefore, they might represent a new class of athero-protective agents (Muse et al., 2018). It has been demonstrated the efficacy of DMHCA and MePipHCA in suppressing inflammation without causing liver lipid accumulation or liver injury in mouse models (Yu et al., 2016) (**Table 1**). Further studies will be needed to evaluate the effect of these compounds on macrophage polarization and activation. A limitation of these compounds is the large dose for *in vivo* efficacy. Therefore, the pharmacokinetic profile of these molecules will need to be improved.

**Table 1:** Overview of nuclear receptors functions modulating lipid metabolism in macrophages and actions mediated by their synthetic ligands.

Receptors	Role in macrophages polarization	Role in lipid metabolism	Agonist or antagonis
PPARγ	Mediates macrophage differentiation via STAT-6 into M2a cells (Huang et al., 1999)	Regulates lipid uptake through the scavenger receptor CD36 (Szanto and Roszer, 2008) Regulates β-oxidation of fatty acids (Vats et al., 2006)	***PPARγ antagonists:*** **GW9662** induces M2c polarizing effects, with upregulation of MerTK (Zizzo and Cohen, 2015) **BZ-26** attenuates inflammation by inhibiting the differentiation inflammatory macrophages (Bei et al., 2016)
LXRs	Promote uptake of ACs through the induction of MerTK (A-Gonzalez et al., 2009)	Act as oxysterols sensors Mediate cholesterol efflux (Szanto and Roszer, 2008)	***LXRs agonists:*** **T0901317** upregulates MerTK expression (Zizzo and Cohen, 2015) (A-Gonzalez et al., 2009) **DMHCA and MePipHCA** act as potent activators of LXRs target genes involved in cholesterol efflux (Magida and Evans, 2018)

## Conclusions and Future Perspectives

Although there is a clear association between NAFLD and the progression of atherosclerotic lesions, the underlying mechanisms are only partially delineated. Lipid metabolism plays key roles in the polarization of macrophages, which, in turn, influences the pathogenesis of lipid-related diseases (**Figure 1**). The huge complexity of NAFLD and CVD pathogenesis suggests a multitarget pharmacological approach, in which macrophages represent an intriguing target.

In this scenario, the nuclear receptor-dependent regulation of MerTK is important for immune homeostasis and MerTK regulates the pro-inflammatory responses reducing both autoimmunity and atherosclerosis.

## Author Contributions

MP, SG, RMP, GL, CR, SP, and FM contributed to analysis of publications, drafting of the manuscript, and critical revision of the content.

## Funding

Funding for this work was partially provided by Italian Foundation of Cancer Research award (MFGA17588) to CR and (IG17786) to FM as well as by Istituto Toscano Tumori (DDRT 6685) to FM.

## Conflicts of Interest Statement

The authors declare that the research was conducted in the absence of any commercial or financial relationships that could be construed as a potential conflict of interest.

## Abbreviations

nonalcoholic fatty liver disease (NAFLD), nonalcoholic steatohepatitis (NASH), MerTK (myeloid-epithelial-reproductive tyrosine kinase), hepatocellular carcinoma (HCC), cardiovascular diseases (CVD), insulin resistance (IR), tumor necrosis factor-α (TNF-α), interleukin-1β (IL-1β), transforming growth factor-β (TGF-β), apoptotic cells (ACs), free fatty acids (FFAs), *de novo* lipogenesis (DNL), sterol regulatory element-binding protein 1c (SREBP-1c), patatin-like phospholipase-3 (PNPLA3), transmembrane 6 superfamily member 2 (TM6SF2), low-density lipoproteins (VLDL), triglycerides (TGs), high-density lipoproteins (HDL), low-density lipoproteins (LDL), growth arrest-specific 6 (Gas-6), carboxyl-glutamic acid (Gla), epidermal growth factor (EGF), sex hormone-binding globulin-like (SHBG), laminin G (LG), visceral adipose tissue (VAT), granulocyte macrophage colony-stimulating factor (GM-CSF), reactive oxygen species (ROS), reactive nitrogen intermediates (RNI), hepatic stellate cells (HSCs), macrophage colony-stimulating factor (M-CSF), growth arrest-specific-6 (Gas-6), matrix metalloproteinases (MMPs), inducible NO synthase (iNOS), hypoxia-inducible factor (HIF), pyruvate kinase M2 (PKM2), peroxisome proliferator-activated receptors (PPARs), liver X receptors (LXRs), retinoid X receptors (RXRs), dendritic cells (DCs), ATP-binding cassette transporter A1 and G1 (ABCA-1 and ABCG-1), reverse cholesterol transport (RCT), apolipoprotein B (APO-B), smooth muscle cells (VSMCs), phosphatidylserine (PtSer), toll-like receptor 4 (TLR4), soluble fragment of MerTK (sol-Mer), specialized pro-resolving mediators (SPMs), suppression of cytokine signaling-1 and 3 (SOCS-1 and SOCS-3), N,N-dimethyl-3β-hydroxycholenamide (DMHCA), methylpiperidinyl-3β-hydroxycholenamide (MePipHCA).
